# Time-History Analysis of Composite Materials with Rectangular Microstructure under Shear Actions

**DOI:** 10.3390/ma14216439

**Published:** 2021-10-27

**Authors:** Marco Colatosti, Nicholas Fantuzzi, Patrizia Trovalusci

**Affiliations:** 1 DISG Department, Sapienza University of Rome, Via A. Gramsci 53, 00197 Rome, Italy; marco.colatosti@uniroma1.it; 2 DICAM Department, University of Bologna, Viale del Risorgimento 2, 40136 Bologna, Italy; nicholas.fantuzzi@unibo.it

**Keywords:** composite materials, multiscale procedures, micropolar continua, time-history analysis

## Abstract

It has been demonstrated that materials with microstructure, such as particle composites, show a peculiar mechanical behavior when discontinuities and heterogeneities are present. The use of non-local theories to solve this challenge, while preserving memory of the microstructure, particularly of internal length, is a challenging option. In the present work, composite materials made of rectangular rigid blocks and elastic interfaces are studied using a Cosserat formulation. Such materials are subjected to dynamic shear loads. For anisotropic media, the relative rotation between the local rigid rotation and the microrotation, which corresponds to the skewsymmetric part of strain, is crucial. The benefits of micropolar modeling are demonstrated, particularly for two orthotropic textures of different sizes.

## 1. Introduction

The most promising types of materials employed in numerous fields of inventive industry are modern composites. Materials such as ceramic [1,2,3,4] and metal composites, poly-crystals (e.g., alumina, zirconia) [5,6,7,8], masonry [9], porous rocks are examples of particle composites: their macroscopic behavior is strongly dependent on the internal microstructure, therefore, discontinuities and heterogeneities cannot be neglected. Detailed modeling is required for an appropriate mechanical description: a discrete model of the microstructure gives a high level of representation, but the drawback is the expensive computational cost [10,11,12,13,14,15] which increases with the scale reduction of the material [16], multiscale approaches are a viable option to derive equivalent homogenized continua [17,18,19,20], however, it has been shown that the classic Cauchy continuum is not reliable for those types of materials when the heterogeneities size has a prominent role, as in the presence of geometric discontinuities or high stress gradients [21,22,23,24,25,26,27,28]. For these reasons, a non-local description is necessary to take into account the microscopic effect on the macroscopic mechanical response. Continuum theories have a non-local character in the presence of internal length parameters (distance between particle in discrete structure, grain or cell size, correlation radius of at-a-distance force, etc.) and spatial dispersion properties (wave velocities depending on wavelength or frequency) [29,30,31,32]. It is worth mentioning that continua with additional degrees of freedom can be considered “implicitly” non-local [29,33].

Thanks to advances in nanostructures and nanotechnologies fields, nanomaterials are progressively commercialized. Materials with structure at the nanoscale generally have peculiar thermophysical and mechanical properties, and non-local theories, of an “explicit” or “implicit” kind, are usually applied to tackle the dynamical behavior of composites such as nanowires [34], nanobeams [35,36], nanorods [37], nanotubes [38,39,40,41], nanoplates [42] and composite beams [43] and plates [44,45].

Nonlocal theories have been used since 19th century (Voigt and Poincaré [29,46]) and applied in the “implicit” and “explicit” from the 1960s and 1970s. A comparison between the two non local approaches, the latter adopted in this paper, have been proposed for instance in [30,31].

The micropolar continuum can be considered as a micromorphic model, where the microdeformation is constrained to be a rotation (microrotation). The Cosserat model is a collection of rigid particles that undergo homogeneous displacements and rotations and interact via forces and couples. Instead, by way of example, for the second gradient and the Cauchy models, the particles are locally constrained to have the same rotation and to interact via forces and moments of forces; in particular, the continuum is a second gradient model if the displacements field is of the second order; while the continuum is a classical model if the field is homogeneous [28].

At different scale levels, micropolar models have already been adopted to describe materials made as an assembly of rigid particles which interacts through elastic interfaces. A typical example are masonry structures where the blocks have rectangular geometry and the mortar joints are modelled as linear elastic springs [23,24,25,26]. The micropolar theory takes into account extra degrees of freedom, which is referred to as microrotation (rotation of a point) to be distinguished from the macrorotation (local rigid rotation). It is worth noting that these rotations coincide in both the couple-stress and classical theories [24]. Micropolar effects become prominent in the presence of load or geometrical singularities, such as concentrated loads, voids or material inclusions. For anisotropic media, the additional strain measure of the so-called relative rotation, defined as the difference between microrotation and macrorotation, corresponding to the skew-symmetric part of the displacement gradient, makes an important contribution to the mechanical behavior [27,28].

In this work, two types of rectangular block textures at three different level scales are considered: the goal is to emphasize the advantages and the necessity of a description of these materials as micropolar continua compared to the classical continua even for dynamic conditions [47] not yet fully examined by the authors. For this purpose, a time-history analysis is used to determine the response of a structure under dynamic load. A homogenization technique, based on an energy equivalence criterion [23,24] between the discrete model, assumed as the benchmark, and the continuum model, is adopted to detect the anisotropic constitutive characteristics [48].

The paper is organized as follows: in Section 2, theoretical background on micropolar continua is presented for the two-dimensional case, in Section 3, the rectangular geometries, the reference volume elements and their relative constitutive properties of materials are reported; in Section 4, the numerical implementation of the structural problem is discussed, in Section 5, a brief review of time transient analysis is reported and the results are discussed and finally the conclusions are summarized in Section 6.

## 2. Micropolar Continuum

Let us consider a linearized kinematical framework. The displacement field for a two-dimensional micropolar continuum is made up of three degrees of freedom, two displacements $u_{1}$, $u_{2}$ and a microrotation ω. In order to use the matrix notation, the displacement vector is defined as $u^{⊤}=\left[\begin{array}{ccc} u_{1} & u_{2} & \omega \end{array}\right]$. The strain and stress vectors are respectively $\varepsilon^{⊤}=\left[\begin{array}{cccccc} \varepsilon_{11} & \varepsilon_{22} & \varepsilon_{12} & \varepsilon_{21} & \kappa_{1} & \kappa_{2} \end{array}\right]$, where the terms $\epsilon_{ij}$ are the normal and tangential strain components, with $\varepsilon_{12}$ and $\varepsilon_{21}$ not equal, and $\kappa_{1}$ and $\kappa_{2}$ are the microcurvatures, and $\sigma^{⊤}=\left[\begin{array}{cccccc} \sigma_{11} & \sigma_{22} & \sigma_{12} & \sigma_{21} & \mu_{1} & \mu_{2} \end{array}\right]$ where the terms $\sigma_{12}$, $\sigma_{21}$ are not equal and the terms $\mu_{1}$, $\mu_{2}$ represent the microcouples.

Defining the operator D:

(1) $$D^{⊤}=\left[\begin{array}{cccccc} \frac{\partial }{\partial x_{1}} & 0 & \frac{\partial }{\partial x_{2}} & 0 & 0 & 0\\ 0 & \frac{\partial }{\partial x_{2}} & 0 & \frac{\partial }{\partial x_{1}} & 0 & 0\\ 0 & 0 & 1 & -1 & \frac{\partial }{\partial x_{1}} & \frac{\partial }{\partial x_{2}} \end{array}\right]$$

is possible to write the kinematic compatibility between the vectors u and ε:

(2) ε=Du

The equilibrium of the body can be expressed using Hamilton’s principle:

(3) $$\delta \int_{t_{1}}^{t_{2}}\left(K-\Pi \right)\;dt=0$$

where *K* is the kinetic energy and Π is the total potential energy given by the sum of the strain energy *U* and the potential of external loads *V*:

(4) Π=U+V

The variation of the kinetic energy is:

(5) $$\delta K=\int_{V}\rho \delta {\dot{u}}^{⊤}\dot{u}\;dV=h\int_{A}\delta {\dot{u}}^{⊤}m\dot{u}\;dA=-h\int_{A}\delta u^{⊤}m\ddot{u}\;dA$$

where *h* is the thickness of the body which can be assumed unitary and m is the equivalent mass matrix defined as:

(6) $$m=\left[\begin{array}{ccc} \rho h & 0 & 0\\ 0 & \rho h & 0\\ 0 & 0 & \rho J_{c} \end{array}\right]$$

where ρ is the material density and $J_{c}$ represents the rotary inertia of the material point. The variation of the strain energy is written in the form:

(7) $$\delta U=\int_{A}\delta \varepsilon^{⊤}\sigma \;dA$$

and using the Equation (1):

(8) $$\delta U=\int_{A}\delta u^{⊤}D^{⊤}\;\sigma \;dA$$

Finally, the variation of the potential of external loads is:

(9) $$\delta V=-\int_{A}\delta u^{⊤}b\;dA-\int_{\gamma_{t}}\delta u^{⊤}t\;d\gamma$$

where the vectors b and t indicate the body and surface forces, respectively.

The micropolar anisotropic constitutive equation takes the form:

(10) σ=Cε

where

(11) $$C=\left[\begin{array}{cccccc} A_{1111} & A_{1122} & A_{1112} & A_{1121} & B_{111} & B_{112}\\ \; & A_{2222} & A_{2212} & A_{2221} & B_{221} & B_{222}\\ \; & \; & A_{1212} & A_{1221} & B_{121} & B_{122}\\ \; & \; & & A_{2121} & B_{211} & B_{212}\\ \; & \; & & \; & D_{11} & D_{12}\\ sym & \; & & \; & & D_{22} \end{array}\right]=\left[\begin{array}{cc} A & B\\ B^{⊤} & D \end{array}\right]$$

The constitutive matrix is symmetric (C∈Sym) when hyperelastic materials are considered: in particular $A_{ijhk}=A_{hkij}$; $B_{ijh}=B_{hij}$; $D_{ij}=D_{ji}$ [25]. From these assumptions, Hamilton’s principle can be written as

(12) $$\int_{t_{1}}^{t_{2}}\left(\int_{A}\delta u^{⊤}\left(m\ddot{u}+D^{⊤}\;CDu\right)dA\;+\int_{A}\delta u^{⊤}b\;dA+\int_{\gamma_{t}}\delta u^{⊤}t\;d\gamma \right)\;dt=0$$

## 3. Rectangular Microstructure

**Figure 1:**
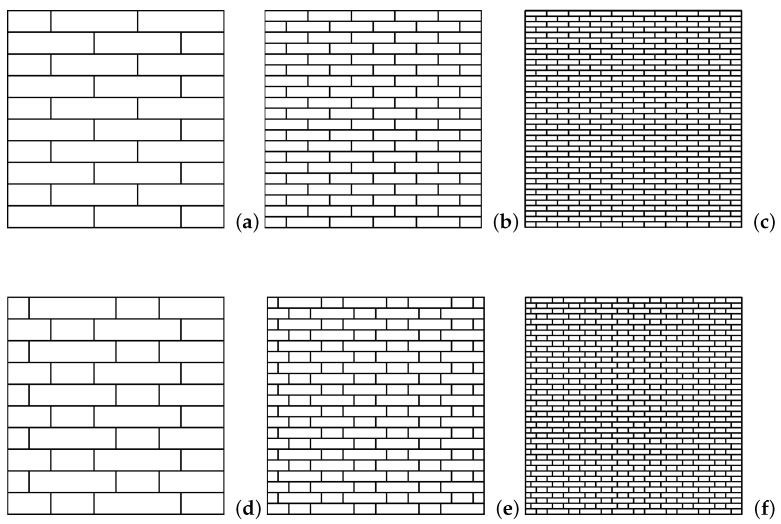
Rectangular microstructures. Texture 1: (**a**) s=1 (**b**) s=0.5 (**c**) s=0.25. Texture 2: (**d**) s=1 (**e**) s=0.5 (**f**) s=0.25.

Materials particle composites with rectangular microstructure are explored in this paper: masonry structures can be assumed as materials of this type. The masonry system is modeled as a discrete system in which the blocks can be considered as rigid bodies and the mortar joints can be assumed as elastic interfaces. Two different types of orthotropic textures that belong to the centrosymmetric class of material have been examined: texture 1 and texture 2 at three different scales (Figure 1). The reference block is 80 cm width and 20 cm high for the scale 1, for texture 2 the smaller block has a half width of the reference block. For the scale s=0.5 and s=0.25, all the geometries’ lengths are obtained by multiplying the dimensions of the scale 1 for the considered scale factor. The aim is to study the dynamic response of the system in a 2D state plane of tension under a shear load modeled as a discrete system, considered as the benchmark, as a micropolar and a classical continuum. Because masonry is a system of rigid elements arranged according to a periodical texture, it possible to define a reference volume element (RVE) from which the constitutive properties of the equivalent continuum can be derived.

### Reference Volume Element

Based on a generalization of the Cauchy–Born rule, starting from a kinematic correspondence map between discrete and continuous fields, an energy equivalence criterion is assumed. To apply the homogenization method to periodic assemblies, the Representative Volume Element (RVE) must first be identified [24].

**Figure 2:**
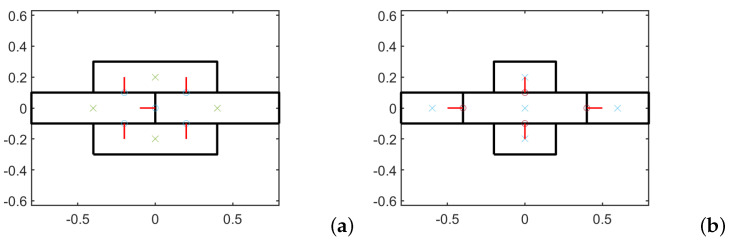
RVEs for the two microstures at scale s=1: (**a**) texture 1 (**b**) texture 2.

The RVEs considered for the two textures are depicted in Figure 2: the RVE of texture 1 is made of four blocks and five elastic links which express the elasticity of bed and head joints; whereas the RVE of texture 2 is made of a central block and four smaller blocks with four elastic links. The material symmetries have to be preserved in the homogenization process [25,49] and as a consequence of the homogenization procedure adopted, it is possible to obtain the constitutive matrices of the materials. The procedure for the springs stiffness calculus of the RVE and the relative constitutive parameters is reported in detail in [28]. In short, homogenization is carried out by considering a RVE with a central block or interface and all the other links in the neighbourhood of such central entity. Elastic springs with normal, shear and rotational components are considered and included.

The constitutive matrices for texture 1 are:

(13) $$A{}_{text1}=10^{11}\left[\begin{array}{cccc} 9.00 & 0.0 & 0 & 0\\ 0.0 & 2.00 & 0 & 0\\ 0 & 0 & 0.80 & 0.0\\ 0 & 0 & 0.0 & 3.60 \end{array}\right]\;D_{text_{1}}^{s=1}=10^{11}\left[\begin{array}{cc} 0.2340 & 0.0\\ 0.0 & 0.0400 \end{array}\right]\;D{}_{text_{1}}^{s=0.5}=10^{11}\left[\begin{array}{cc} 0.0585 & 0\\ 0 & 0.0100 \end{array}\right]\;D_{text_{1}}^{s=0.25}=10^{11}\left[\begin{array}{cc} 0.0146 & 0\\ 0 & 0.0025 \end{array}\right]$$

The constitutive matrices for texture 2 are:

(14) $$A{}_{text_{2}}=10^{12}\left[\begin{array}{cccc} 1.92 & 0.17 & 0 & 0\\ 0.17 & 0.20 & 0 & 0\\ 0 & 0 & 0.08 & 0.0\\ 0 & 0 & 0.0 & 1.44 \end{array}\right]\;D_{text_{2}}^{s=1}=10^{12}\left[\begin{array}{cc} 0.0610 & 0.0015\\ 0.0015 & 0.0112 \end{array}\right]\;D{}_{text_{2}}^{s=0.5}=10^{12}\left[\begin{array}{cc} 0.0153 & 0.0004\\ 0.0004 & 0.0028 \end{array}\right]\;D_{text_{2}}^{s=0.25}=10^{12}\left[\begin{array}{cc} 0.0038 & 0.0001\\ 0.0001 & 0.0007 \end{array}\right]$$

and, both textures being centrosymmetric, B=0 for both textures. The internal length of the material is taken into account by the sub matrix D where, approximately, $D_{s=1}\approx 4D_{s=0.5}\approx 16D_{s=0.25}$. Note that the elements’ size affects only the matrix D.

The constitutive models for the Cauchy continuum are obtained from the previous constitutive matrices [27] as:

$$C=\left[\begin{array}{ccc} A_{1111} & A_{1122} & 0\\ A_{2211} & A_{2222} & 0\\ 0 & 0 & \frac{1}{2}\left[A_{1212}+A_{2121}\right]+A_{1221} \end{array}\right]$$

The classical model does not preserve a memory of the internal length of the microstructure.

## 4. Numerical Implementation

The differential equation problem for the classical and micropolar models are both solved through a MATLAB finite element method (FEM) code [50]. Newmark’s method [51] is also implemented to investigate the dynamic response of a wall subjected to a dynamic shear load and the results are compared with a discrete model prepared with the FEM software ABAQUS (Dassault Systèmes, Johnston, RI, USA).

### Continuum Model

To solve the continuum numerical problem, a mesh of 32 × 32 elements Q4 finite element with reduced integration is employed. The numerical problem is solved in terms of displacements and in order to apply reduced integration, the strain vector has to be rearranged by separating strain terms which are fully integrated and the ones for which reduced integration is applied [49,52].

The finite element method is based on the approximation of nodal displacements:

(15) $$u=N\;d^{e}$$

The kinematic displacement vector is arranged as follows:

(16) $$d^{eT}=\left[\begin{array}{ccccccccc} u_{1}^{1} & \ldots & u_{1}^{4} & u_{2}^{1} & \ldots & u_{2}^{4} & \omega^{1} & \ldots & \omega^{4} \end{array}\right]$$

with 12 degrees of freedom overall (3 per node). The matrix of the shape functions is composed by the vector N that collects the Lagrangian shape functions:

(17) $$N=\left[\begin{array}{ccc} N & 0 & 0\\ 0 & N & 0\\ 0 & 0 & N \end{array}\right]$$

Including the above expression in the Hamilton principle, the kinetic energy becomes:

(18) $$\delta K=-\delta d^{eT}\int_{A}N^{⊤}mN\;dA\;{\ddot{d}}^{\;e}$$

The mass matrix reads:

(19) $$M^{e}=\int_{A}N^{⊤}mN\;dA$$

The internal work takes the form:

(20) $$\delta U=\delta d^{eT}h\int_{A}{\left(D\;N\right)}^{⊤}C\left(D\;N\right)\;dA\;d^{e}=\delta d^{eT}h\int_{A}B^{⊤}C\;B\;dA\;d^{e}$$

where B=DN, thus, the element stiffness matrix is:

(21) $$K^{e}=\int_{A}B^{⊤}C\;B\;dA$$

which has to be integrated according to a 2×2 Gauss integration for the normal components as well as microcouples, whereas reduced integration is applied on shear components. Finally, the potential of external forces is:

(22) $$\delta V=-\delta {d^{e}}^{T}\int_{A}N^{⊤}b\;dA-\delta {d^{e}}^{T}\int_{\gamma_{t}}N^{⊤}t\;d\gamma =-\delta {d^{e}}^{T}F$$

where F is the global vector of volume and surface forces.

## 5. Simulations

**Figure 3:**
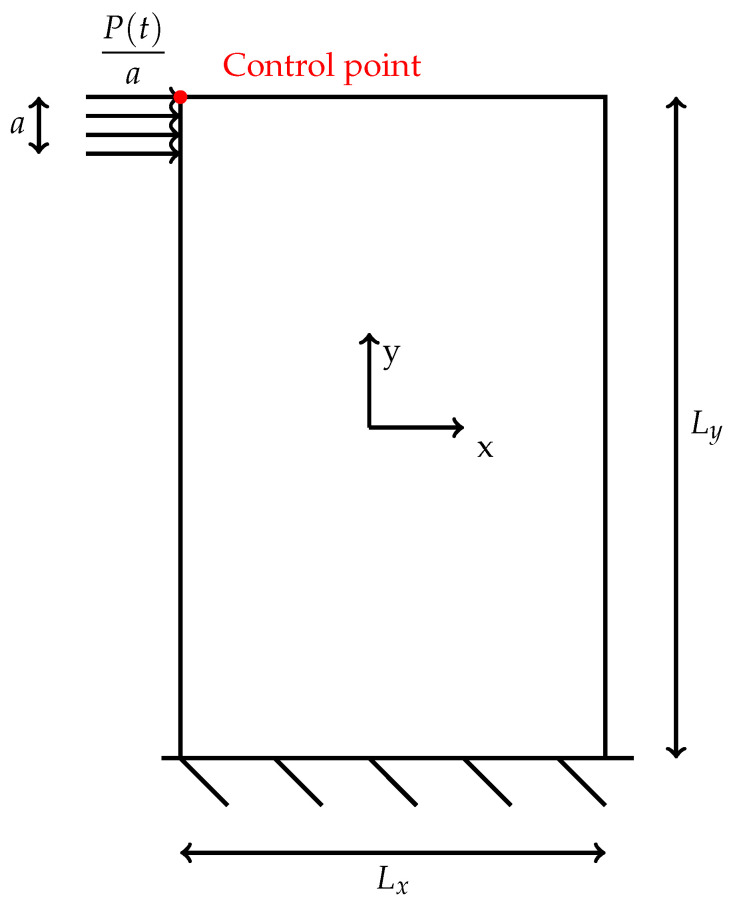
Schematic of the panel analyzed.

The results reported below investigate the behavior of a wall, clamped at the base and subjected to a distributed dynamic shear load applied at the top (Figure 3), in order to extend the numerical results already obtained for the static case [48] and to enrich the aspects related to the dynamic conditions [47]. Furthermore, a numerical evaluation, for the case in which blocks of different sizes are present in the masonry texture, wants to be performed, because in previous works, this aspect has been evaluated only in qualitative terms [25]. The footprint of the load is equal to $a=L_{y}/8$, where $L_{y}$ is the height of the panel and $L_{x}$ is the width. The data results are reported in terms of displacements; in particular, the displacements of the control point as a function of time are plotted for three different mechanical models: discrete, micropolar and classical. For texture 1, the panel has dimensions $L_{x}=3.2$ m and $L_{y}=4$ m, whereas for texture 2 the dimensions are $L_{x}=3$ m and $L_{y}=3.2$ m. The expression of the horizontal dynamic load is:

(23) $$P\left(t\right)=q_{0}\left(1-\cos\left(ft\right)\right);$$

where $q_{0}=100$ kN, the time domain is equal to 1000 s, the time step is equal to 5 s and the angular frequency f=0.02 Hz (load case 1). Finally, further numerical simulations were carried out considering the same load type but with a frequency value equal to the frequency of the first free vibration mode of the structure in a time period of 0.01 (load case 2).

### 5.1. Time Transient Analysis

The Newmark method is briefly reported in [50]. The equation that must be solved is:

(24) $$M\ddot{d}+Kd=F$$

where M is the mass matrix, K is the stiffness matrix, d is the displacement vector and F is the vector of the external loads, with the initial conditions d=0 and $\dot{d}=0$ at time t=0. The time functions are approximated by Taylor’s series arrested at the second-order derivative. The time increment is indicated as $dt=t_{s+1}-t_{s}$. The velocity and the acceleration vector can be written as

(25) $${\dot{d}}_{s+1}={\dot{d}}_{s}+a_{1}{\ddot{d}}_{s}+a_{2}{\dot{d}}_{s+1}$$

(26) $${\ddot{d}}_{s+1}=a_{3}\left(d_{s+1}-d_{s}\right)-a_{4}{\dot{d}}_{s}-a_{5}{\ddot{d}}_{s}$$

The coefficients are

(27) $$a_{1}=\left(1-\alpha \right)\;dt,\;\;\;a_{2}=\alpha \;dt,\;\;\;a_{3}=2/\gamma \;dt^{2},\;\;\;a_{4}=a_{3}\;dt,\;\;\;a_{5}=\left(1-\gamma \right)/\gamma$$

The parameters α and γ depend on the time integration scheme. For this study case, the constant average acceleration method has been used, thus, α=1/2, γ=1/2. The algebraic system of equations at the generic time $t_{s+1}$ becomes:

(28) $$\hat{K}d_{s+1}=\hat{F},\;\;d_{s+1}={\hat{K}}^{-1}\hat{F}$$

where:

(29) $$\hat{K}=K_{s+1}+a_{3}M_{s+1}$$

(30) $$\hat{F}=F_{s+1}+M_{s+1}\left(a_{3}d_{s}+a_{4}{\dot{d}}_{s}+a_{5}{\ddot{d}}_{s}\right)$$

All d quantities are known at the time $t_{s}$. The mass matrix, M, and stiffness, K, matrix remain constant. By using starting values for displacement and velocities at time t=0, the initial acceleration can be carried out as:

(31) $$\ddot{d}=M_{0}^{-1}\left(F_{0}-K_{0}d_{0}\right)$$

### 5.2. Texture 1

**Figure 4:**
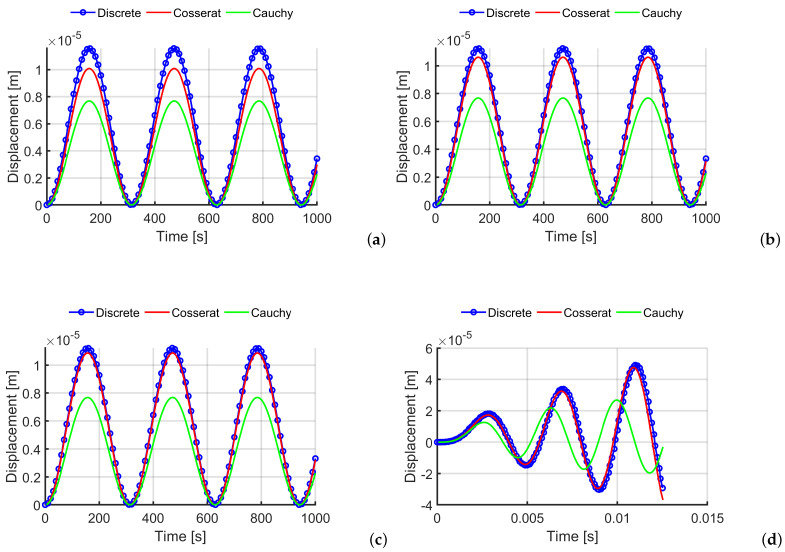
Time-history analysis for texture 1: horizontal displacements for the three texture scales (**a**) s=1 (**b**) s=0.5 (**c**) s=0.25 (load case 1) (**d**) mechanical resonance for the scale s=0.5 (load case 2).

In Figure 4a–c, the control point displacements along the *x* direction are reported for the load case of Equation (23). The graphs depict three scales and three models: Figure 4a shows that both continuum models catch the same trend of the discrete model due the elasticity hypothesis, however, the Cauchy model underestimates the maximum displacement magnitude—in fact, the error is around 33%. In contrast, the Cosserat model shows greater accuracy in evaluating the values (around 12.78% for the first scale, until reaching approximately 3% for the smaller scale), therefore, the micropolar model converges to a discrete model with the scale reduction, whereas the classical continuum does not involve any improvement because it does not take into account the internal length of the material microstructure. Figure 4d shows the displacement of the control point (for the scale s=0.5) at the resonance frequency and the differences between the continuum models are more evident: only the Cosserat model reproduces the result of the discrete system, whereas the Cauchy model presents a response with a phase shift and a gross estimation of the maximum amplitude.

### 5.3. Texture 2

**Figure 5:**
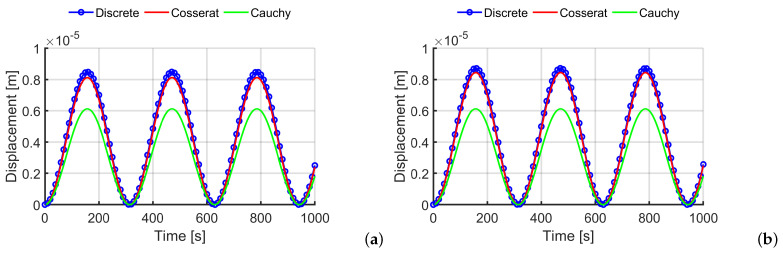

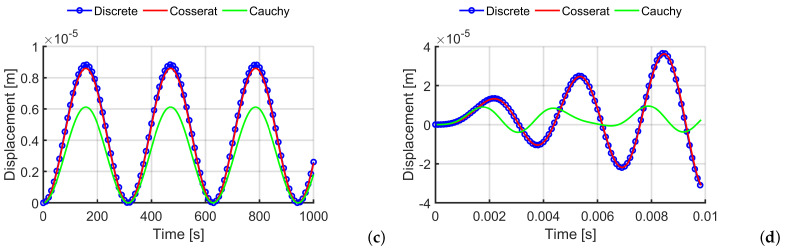
Time-history analysis for texture 2: horizontal displacements for the three texture scales (**a**) s=1; (**b**) s=0.5; (**c**) s=0.25 (load case 1); (**d**) mechanical resonance for the scale s=0.5 (load case 2).

In Figure 5a–c, the horizontal displacements of the control point of the first load case are reported. For texture 2, the Cosserat model provides results with a very good approximation from the first scale s=1, in fact, the error is around 4% for the larger scale and goes below 3% for the smaller one; instead, for the Cauchy model the maximum displacement evaluation gives an error around 30%. Once again, the difference between the discrete and the micropolar model improves by reducing the scale and this is very important since the smaller scale is the one that involves the highest computational burden for discrete models. In Figure 5, the displacement at resonance for the scale s=0.5 is reported. The micropolar model matches with a good quality with the discrete system trend, whereas the classical model is not able to reproduce the response, there is no growth of the displacement values with the time increment.

## 6. Conclusions

This work investigates the dynamic response of particle composites with two different rectangular textures of the microstructure at three different scales. The usefulness of using continuous models for the representation of a complex material is well known, however, the goodness of the results strongly depends on the continuum theory adopted: the numerical analyses prove that the Cauchy continuum is not sufficient to describe the mechanical behavior of microstructured materials, for both textures and for all scales. On the other hand, the Cosserat model, which takes into account the scale effect, is accurate enough to reproduce the response of the discrete system, assumed as the benchmark of the problem, not only in static condition, but even under dynamic forced oscillations. Moreover, in the case of mechanical resonance, the differences are more noticeable: the micropolar continuum showed itself to be always reliable, whereas for the classical model a delay of the response is shown for texture 1 and the tendency of a mechanical system to respond at greater amplitude when the frequency of its oscillations matches the system natural frequency of vibration is completely missed for texture 2. The significant mechanical response discrepancy, between the classical and the micropolar model, may be related to the fact that for this microstructure, two blocks of different sizes were considered and the internal length plays an important role. Since the off-diagonal terms ($D_{12}$ and $D_{21}$) are non-zero, the micropolar contribution is remarkable, as seen for anisotropic materials, and this explains the major differences with the Cauchy model. Classical theory is not accurate enough to properly describe materials where the internal length has a prominent influence such as masonry, even if the simulations are limited to the elastic case only. In view of all these further analyses, which have not yet been addressed by the authors, new research will be conducted, considering nonlinear mechanical conditions such as crack and damage, as well as structural damping, for a more complete and exhaustive analysis of microstructured materials. Combining these last results with earlier analyses, it can be acknowledged that the Cosserat continuum is able to properly describe the response of the discrete system which is assumed as a micromodel for masonry-like systems.

## Data Availability

Some or all data, models, or code that support the findings of this study are available from the corresponding author upon reasonable request.

## References

[1] Sadowski T., Samborski S. (2003). Prediction of the mechanical behaviour of porous ceramics using mesomechanical modelling. Comput. Mater. Sci..

[2] Sadowski T., Samborski S. (2008). Development of damage state in porous ceramics under compression. Comput. Mater. Sci..

[3] Samborski S., Sadowski T. (2010). Dynamic fracture toughness of porous ceramics. J. Am. Ceram. Soc..

[4] Postek E., Sadowski T. (2011). Assessing the influence of porosity in the deformation of metal–ceramic composites. Compos. Interfaces.

[5] Sadowski T., Postek E., Denis C. (2007). Stress distribution due to discontinuities in polycrystalline ceramics containing metallic inter-granular layers. Comput. Mater. Sci..

[6] Sadowski T., Marsavina L. (2011). Multiscale modelling of two-phase ceramic matrix composites. Comput. Mater. Sci..

[7] Boniecki M., Sadowski T., Gołębiewski P., Węglarz H., Piątkowska A., Romaniec M., Krzyżak K., Łosiewicz K. (2020). Mechanical properties of alumina/zirconia composites. Ceram. Int..

[8] Sadowski T., Łosiewicz K., Boniecki M., Szutkowska M. (2021). Assessment of mechanical properties by nano-and microindentation of alumina/zirconia composites. Mater. Today Proc..

[9] Gatta C., Addessi D., Vestroni F. (2018). Static and dynamic nonlinear response of masonry walls. Int. J. Solids Struct..

[10] Addessi D., Sacco E. (2016). Enriched plane state formulation for nonlinear homogenization of in-plane masonry wall. Meccanica.

[11] Addessi D., Sacco E. (2018). Homogenization of heterogeneous masonry beams. Meccanica.

[12] Baggio C., Trovalusci P. (2000). Collapse behaviour of three-dimensional brick-block systems using non-linear programming. Struct. Eng. Mech..

[13] Godio M., Stefanou I., Sab K., Sulem J., Sakji S. (2017). A limit analysis approach based on Cosserat continuum for the evaluation of the in-plane strength of discrete media: Application to masonry. Eur. J. -Mech.-A/Solids.

[14] Baraldi D., Reccia E., Cecchi A. (2018). In plane loaded masonry walls: DEM and FEM/DEM models. A critical review. Meccanica.

[15] Reccia E., Leonetti L., Trovalusci P., Cecchi A. (2018). A multiscale/multidomain model for the failure analysis of masonry walls: A validation with a combined FEM/DEM approach. Int. J. Multiscale Comput. Eng..

[16] Pepe M., Pingaro M., Trovalusci P., Reccia E., Leonetti L. (2020). Micromodels for the in-plane failure analysis of masonry walls: Limit Analysis, FEM and FEM/DEM approaches. Frat. Integrità Strutt..

[17] Trovalusci P., Ostoja-Starzewski M. (2011). Multiscale mechanical modelling of complex materials and engineering applications 2. Int. J. Multiscale Comput. Eng..

[18] Sadowski T., Trovalusci P., Schrefler B., de Borst R. (2014). Multi-scale and multi-physics modelling for complex materials. Meccanica.

[19] Altenbach H., Sadowski T. (2015). Failure and Damage Analysis of Advanced Materials.

[20] Greco F., Leonetti L., Luciano R., Blasi P.N. (2016). Effects of microfracture and contact induced instabilities on the macroscopic response of finitely deformed elastic composites. Compos. Part Eng..

[21] De Borst R. (1991). Simulation of Strain Localization: A Reappraisal of the Cosserat Continuum. Eng. Comp..

[22] Sluys L., De Borst R., Mühlhaus H.B. (1993). Wave propagation, localization and dispersion in a gradient-dependent medium. Int. J. Solids Struct..

[23] Masiani R., Rizzi N., Trovalusci P. (1995). Masonry as structured continuum. Meccanica.

[24] Masiani R., Trovalusci P. (1996). Cosserat and Cauchy materials as continuum models of brick masonry. Meccanica.

[25] Trovalusci P., Masiani R. (1999). Material symmetries of micropolar continua equivalent to lattices. Int. J. Solids Struct..

[26] Trovalusci P., Masiani R. (2005). A multifield model for blocky materials based on multiscale description. Int. J. Solids Struct..

[27] Pau A., Trovalusci P. (2012). Block masonry as equivalent micropolar continua: The role of relative rotations. Acta Mech..

[28] Trovalusci P., Pau A. (2014). Derivation of microstructured continua from lattice systems via principle of virtual works: The case of masonry-like materials as micropolar, second gradient and classical continua. Acta Mech..

[29] Trovalusci P., Sadowski T., Trovalusci P. (2014). Molecular Approaches for Multifield Continua: Origins and current developments. Multiscale Modeling of Complex Materials: Phenomenological, Theoretical and Computational Aspects.

[30] Tuna M., Trovalusci P. (2020). Scale dependent continuum approaches for discontinuous assemblies: ‘Explicit’ and ‘implicit’ non-local models. Mech. Res. Commun..

[31] Tuna M., Leonetti L., Trovalusci P., Kirca M. (2020). ‘Explicit’ and ‘implicit’ non-local continuous descriptions for a plate with circular inclusion in tension. Meccanica.

[32] Tuna M., Trovalusci P. (2021). Stress distribution around an elliptic hole in a plate with ‘implicit’ and ‘explicit’ non-local models. Compos. Struct..

[33] Eringen A.C. (1999). Theory of micropolar elasticity. Microcontinuum Field Theories.

[34] Uzun B., Civalek Ö. (2019). Nonlocal FEM formulation for vibration analysis of nanowires on elastic matrix with different materials. Math. Comput. Appl..

[35] Barretta R., Luciano R., de Sciarra F.M. (2015). A Fully Gradient Model for Euler-Bernoulli Nanobeams. Math. Probl. Eng..

[36] Čanađija M., Barretta R., De Sciarra F.M. (2016). A gradient elasticity model of Bernoulli–Euler nanobeams in non-isothermal environments. Eur. J.-Mech.-A/Solids.

[37] Barretta R., Faghidian S.A., Luciano R. (2019). Longitudinal vibrations of nano-rods by stress-driven integral elasticity. Mech. Adv. Mater. Struct..

[38] Barretta R., Marotti de Sciarra F. (2013). A Nonlocal Model for Carbon Nanotubes under Axial Loads. Adv. Mater. Sci. Eng..

[39] Eshraghi I., Jalali S.K., Pugno N.M. (2016). Imperfection sensitivity of nonlinear vibration of curved single-walled carbon nanotubes based on nonlocal timoshenko beam theory. Materials.

[40] Izadi R., Tuna M., Trovalusci P., Ghavanloo E. (2021). Torsional characteristics of carbon nanotubes: Micropolar elasticity models and molecular dynamics simulation. Nanomaterials.

[41] Civalek Ö., Dastjerdi S., Akbaş Ş.D., Akgöz B. (2021). Vibration analysis of carbon nanotube-reinforced composite microbeams. Math. Methods Appl. Sci..

[42] Tocci Monaco G., Fantuzzi N., Fabbrocino F., Luciano R. (2021). Trigonometric solution for the bending analysis of magneto-electro-elastic strain gradient nonlocal nanoplates in hygro-thermal environment. Mathematics.

[43] Akbaş Ş.D., Ersoy H., Akgöz B., Civalek Ö. (2021). Dynamic Analysis of a Fiber-Reinforced Composite Beam under a Moving Load by the Ritz Method. Mathematics.

[44] Addessi D. (2014). A 2D Cosserat finite element based on a damage-plastic model for brittle materials. Comput. Struct..

[45] Bacciocchi M., Tarantino A. (2019). Natural Frequency Analysis of Functionally Graded Orthotropic Cross-Ply Plates Based on the Finite Element Method. Math. Comput. Appl..

[46] Capecchi D., Ruta G., Trovalusci P. (2011). Voigt and Poincaré’s mechanistic–energetic approaches to linear elasticity and suggestions for multiscale modelling. Arch. Appl. Mech..

[47] Colatosti M., Fantuzzi N., Trovalusci P. (2021). Dynamic Characterization of Microstructured Materials Made of Hexagonal-Shape Particles with Elastic Interfaces. Nanomaterials.

[48] Colatosti M., Fantuzzi N., Trovalusci P., Masiani R. (2021). New insights on homogenization for hexagonal-shaped composites as Cosserat continua. Meccanica.

[49] Fantuzzi N., Trovalusci P., Luciano R. (2020). Material Symmetries in Homogenized Hexagonal-Shaped Composites as Cosserat Continua. Symmetry.

[50] Ferreira A., Fantuzzi N. (2020). MATLAB Codes for Finite Element Analysis 2nd Edition: Solids and Structures.

[51] Newmark N.M. (1959). A method of computation for structural dynamics. J. Eng. Mech. Div..

[52] Fantuzzi N., Trovalusci P., Luciano R. (2020). Multiscale analysis of anisotropic materials with hexagonal microstructure as micropolar continua. Int. J. Multiscale Comput. Eng..

